# Systematic review and meta-analysis of Guipi Decoction in the treatment of anemia

**DOI:** 10.1097/MD.0000000000043625

**Published:** 2025-07-25

**Authors:** Xueyan Lv, Dongmei Wang, Xiangni Zou

**Affiliations:** a Heilongjiang University of Chinese Medicine, Harbin, China; b First Affiliated Hospital of Heilongjiang University of Chinese Medicine, Harbin, China.

**Keywords:** anemia, blood deficiency, Guipi Decoction, meta-analysis, traditional Chinese medicine

## Abstract

**Background:**

Anemia is a common and widely influential public health problem worldwide. The objective was to conduct a comprehensive and in-depth systematic evaluation of the therapeutic efficacy of Guipi Decoction as an adjuvant treatment for patients with anemia and to offer new ideas for the clinical treatment of anemia.

**Methods:**

A comprehensive and systematic literature search was meticulously executed across multiple authoritative databases, including CNKI, Wanfang, VIP, EMBase, Scopus, Web of Science, PubMed, and the Cochrane Library. The search was designed to identify all relevant randomized controlled trials that investigated the application of Guipi Decoction as an adjuvant therapy for Anemia patients. Considering that 1 researcher had written article of this kind in 2020, so the time frame of the search spanned from January 2020 to January 2025. Two independent reviewers selected eligible trials, extracted data, and assessed methodological quality using predefined criteria. Meta-analysis was performed using the standardized software RevMan 5.3.

**Results:**

A total of 13 studies were included in the final analysis, involving a cumulative sample size of 954 participants. These participants were randomly allocated to the experimental group and the control group. Pretreatment physiological parameters were comparable between the 2 groups, with no statistically significant differences, indicating a balanced randomization. Posttreatment, the experimental group demonstrated significantly better outcomes across multiple clinically relevant endpoints compared to the control group. The indicators include the effective rate, adverse event rate, hemoglobin, red blood cell, serum iron, serum ferritin, and hematocrit. For the experimental group, there are statistically significant differences in various indicators before and after treatment. At the same time, when compared with the indicators of the control group after treatment, the outcome indicators of the experimental group show a better therapeutic effect, and the differences are statistically significant. This can strongly prove that Guipi Decoction has a definite therapeutic effect on treating anemia.

**Conclusion:**

Guipi Decoction can effectively relieve symptoms in anemia patients and reduce risk indicators. It is worthy of further research and promotion.

## 1. Introduction

Anemia, a global public health issue, significantly impacts human health.^[1,2]^ In Western medicine, anemia is defined as a common syndrome where the volume of red blood cells in the human peripheral blood is below the lower limit of the normal range. Red blood cells play a crucial role in transporting oxygen in the body. When anemia occurs, the decrease in the number of red blood cells or abnormalities in their function lead to insufficient oxygen delivery, triggering a series of uncomfortable symptoms.

From a pathophysiological perspective, Western medicine believes that the causes of anemia are complex and diverse. Abnormal hematopoietic stem cells can lead to aplastic anemia; deficiency of hematopoietic raw materials such as iron, vitamin B12, and folic acid can cause iron-deficiency anemia and megaloblastic anemia^[3–5]^; defects in the structure or enzymes of red blood cells themselves can result in hemolytic anemias such as hereditary spherocytosis and glucose-6-phosphate dehydrogenase deficiency; in addition, chronic diseases, autoimmune disorders, and excessive blood loss are also common causes of anemia.^[6]^

Western medicine has a rigorous diagnostic system for anemia.^[7]^ Through multiple examinations such as blood routine, bone marrow aspiration, iron metabolism index tests, and determination of vitamin B12 and folic acid levels, it can accurately determine the type and cause of anemia, providing a basis for subsequent precise treatment. In terms of treatment, Western medicine takes targeted measures according to different causes, such as supplementing iron agents for iron-deficiency anemia, supplementing vitamin B12 and folic acid for megaloblastic anemia, and using immunosuppressants for autoimmune hemolytic anemia.^[8]^ Although Western medicine has made remarkable progress in the diagnosis and treatment of anemia, there are still some limitations. The treatment effects of some types of anemia are not satisfactory, and long-term use of medications may cause adverse reactions. Therefore, exploring more effective treatment methods for anemia has always been an important topic in the medical field.

Traditional Chinese medicine has made significant contributions to the treatment of anemia.^[9]^ In traditional Chinese medicine, it is believed that “the spleen is the foundation of acquired constitution and the source of qi and blood generation.” Guipi Decoction contains herbs such as ginseng, atractylodes macrocephala, and astragalus membranaceus, which play a substantial role in assisting the treatment of anemia.^[10]^ These herbs can invigorate the spleen and replenish qi, promoting the transportation and transformation functions of the spleen and stomach. As a result, the nutrients from food can be better converted into qi and blood, providing a material basis for hematopoiesis. In recent years, Guipi Decoction has been widely used in the clinical treatment of anemia.

## 2. Information and methodology

### 2.1. Sources

#### 2.1.1. Literature inclusion criteria

① Studies must be Randomized Controlled Trials focusing on the adjuvant treatment of anemia using Guipi Decoction, with complete documentation.
② Studies must clearly report diagnostic methods and criteria for anemia.
③ Experimental and control groups must be comparable in terms of baseline characteristics.

#### 2.1.2. Exclusion criteria

① Scientific research findings or patent reports that only have abstracts but no full-text.
② Duplicate reports in the literature.
③ Literature for which, after retrieving the full-text, the data is incomplete or the endpoint outcome is not specified.

### 2.2. Methodology

#### 2.2.1. Search strategy

Search terms included: “Anemia,” “Blood deficiency,” “Guipi Tang,” “Guipi Decoction,” “randomized controlled trial,” and “RCT.” Searches were conducted using a combination of subject headings and free text terms. Major domestic and international academic databases such as CNKI, Wanfang, VIP, PubMed, EMBase, Scopus, Cochrane Library, and Web of Science were searched for RCTs on the adjuvant treatment of anemia patients with Guipi Decoction. Reference lists of included RCTs were also reviewed. The search timeframe was from the inception of each database to January 2025.

#### 2.2.2. Literature screening and data extraction

For the initially retrieved literature, Zotero is used to establish a database. Two researchers independently screened titles and abstracts, excluded duplicates, and excluded studies that did not meet inclusion criteria. Full texts of potentially eligible studies were retrieved and reviewed. Both researchers cross-checked the final list of included studies, conducted quality assessments, and resolved any discrepancies through consultation with a third researcher. Extracted data included publication date, inclusion and exclusion criteria, general information about participants in both trial and control groups, interventions, and outcome measures. For RCTs with incomplete follow-up data, the original articles were reviewed to determine the reasons for data loss.

#### 2.2.3. Literature quality evaluation

Two researchers will assess the quality of included RCTs using the “Cochrane Collaboration’s Risk of Bias Assessment Criteria.” Specific evaluation items include: random sequence generation, allocation concealment, blinding of participants and personnel, blinding of outcome assessment, completeness of outcome data, and selective reporting.

#### 2.2.4. Outcome measures

① Effective rate
② Adverse event rate
③ Hemoglobin (Hb)
④ Red Blood Cell (RBC)
⑤ Serum Iron (SI)
⑥ Serum Ferritin (SF)
⑦ Hematocrit (HCT)

### 2.3. Statistical analysis

Meta-analysis was conducted using RevMan 5.3 software. Continuous data were evaluated using mean difference (MD), and categorical data were evaluated using relative risk (RR). Heterogeneity was assessed using the *I*^2^ test. For *I*^2^ < 25%, indicating no heterogeneity, and a fixed-effect model was applied. For 25% ≤ *I*^2^ < 50%, heterogeneity was considered low, still indicating no significant differences, and a fixed-effect model was applied. For 50% ≤ *I*^2^ < 75%, heterogeneity was considered moderate, and for *I*^2^ ≥ 75%, heterogeneity was considered high, both indicating significant differences, and a random-effects model was applied. Sensitivity analyses and subgroup analyses were performed if feasible to explore sources of heterogeneity. A funnel plot was used to assess publication bias.

## 3. Results

### 3.1. Literature search results

**Figure 1.**
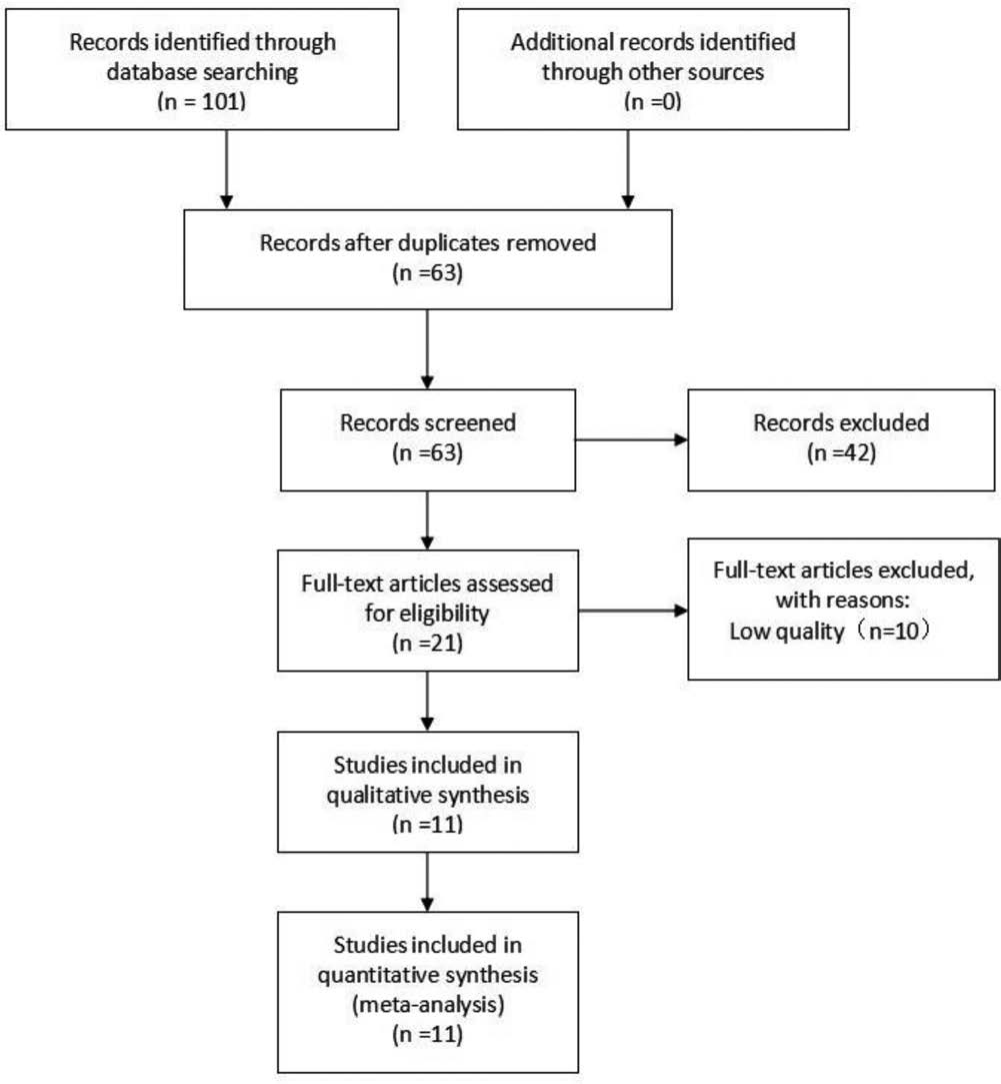
The literature screening process.

A total of 101 documents were initially identified. After excluding duplicates and studies that did not meet the inclusion criteria, 11 articles were finally included following a thorough review of titles, abstracts, and full texts. These studies involved a total of 954 patients, with 480 in the control group and 474 in the experimental group. Eleven literatures have achieved randomized grouping. But, only 2 literatures mention the concealment of the allocation scheme, and the overall quality is mediocre. The literature screening process is illustrated in Figure 1, and the basic information of the included studies is summarized in Table 1.

**Table 1:** Basic characteristics of the 11 studies included in the meta-analysis.

First author	Year of publication	Integrate into number of examples		Age (years)		Intervention		Conclusion norm
		Test group	Control subjects	Test group	Control subjects	Test group	Control subjects	
Du	2020	31	31	42 ± 9.28	41 ± 10.62	Ferrous Sulfate Tablets, 0.3 g, po, tid + vitamin C, 0.2 g, po, tid + Guipi Decoction and Danggui Buxue Decoction, 100 mL, po, bid, course of treatment 60 d	Ferrous Sulfate Tablets, 0.3 g, po, tid + vitamin C, 0.2 g, po, tid, course of treatment 60 d	①②③④⑤⑥
Wu	2023	30	37	23–68	19–68	Ferrous Succinate Sustained-release Tablets, po + Guipi Decoction, po, bid, course of treatment 28 d	Ferrous Succinate Sustained-release Tablets, po + placebo, po, bid, course of treatment 28 d	③④⑤⑥⑦
Liu	2020	48	48	33.02 ± 10.7	33.52 ± 11.74	Symptomatic treatment + Guipi Decoction, 200 mL, po, bid, course of treatment 28 d	Symptomatic treatment, course of treatment 28 d	③④
Chen	2024	65	65	47.07 ± 6.66	46.80 ± 6.25	Symptomatic treatment + Ferrous Succinate Tablets, 0.1 g, po, tid + Guipi Decoction, 150 mL, po, bid, course of treatment 28 d	Symptomatic treatment + Ferrous Succinate Tablets, 0.1 g, po, tid, course of treatment 28 d	①③④⑥
Bao	2023	35	35	43.23 ± 11.37	42.25 ± 10.56	Iron Polysaccharide Complex Capsules, 150 mg, po, qd + Roxadustat Capsules, 100 mg, po, tiw + Guipi Decoction, 100 mL, po, bid, course of treatment 14 d	Iron Polysaccharide Complex Capsules, 150 mg, po, qd + Roxadustat Capsules, 100 mg, po, tiw, course of treatment 14 d	③④
Wen	2023	30	30	63.70 ± 7.25	63.57 ± 7.33	Symptomatic treatment + EPO, 150 IU/kg, H, tiw + Guipi Decoction, 100 mL, po, bid, course of treatment 56 d	Symptomatic treatment + EPO, 150 IU/kg, H, tiw, course of treatment 56 d	①③④
Li	2021	37	37	36.83 ± 13.06	37.02 ± 13.33	Symptomatic treatment + vitamin C, 200 mg, po, tid + Ferrous Sulfate Tablets, 200 mg, po, tid + Guipi Decoction, 150 mL, po, bid	Symptomatic treatment + vitamin C, 200 mg, po, tid + Ferrous Sulfate Tablets, 200 mg, po, tid	①②③⑥
Qiu	2021	60	60	58.54 ± 3.64	59.64 ± 3.73	Symptomatic treatment + EPO, H, qw + Guipi Decoction, po, bid, course of treatment 56 d	Symptomatic treatment + EPO, 10,000 IU, H, qw, course of treatment 56 d	②③⑦
Wang	2020	30	30	63.73 ± 6.38	63.40 ± 8.34	Symptomatic treatment + EPO, 10,000 IU, H, tiw + Guipi Decoction, 200 mL, po, bid, course of treatment 28 d	Symptomatic treatment + EPO, 10,000 IU, H, tiw, course of treatment 28 d	①③④⑦
Li	2020	28	27	62.39 ± 9.44	62.52 ± 11.11	EPO, 10,000 IU, H, biw + Guipi Decoction, 200 mL, po, bid, course of treatment 28 d	EPO, 10,000 IU, H, biw, course of treatment 28 d	①③④⑦
Bao	2024	80	80	Average 60	Average 60	Symptomatic treatment + EPO, 150 µg/kg, H, biw + Guipi Decoction, 200 mL, po, bid, course of treatment 14 d	Symptomatic treatment + EPO, 150 µg/kg, H, biw, course of treatment 14 d	①③④⑦

① Effective rate, ② adverse event rate, ③ hemoglobin, ④ red blood cell, ⑤ serum iron, ⑥ serum ferritin, ⑦ hematocrit.

### 3.2. Literature quality evaluation

**Figure 2.**
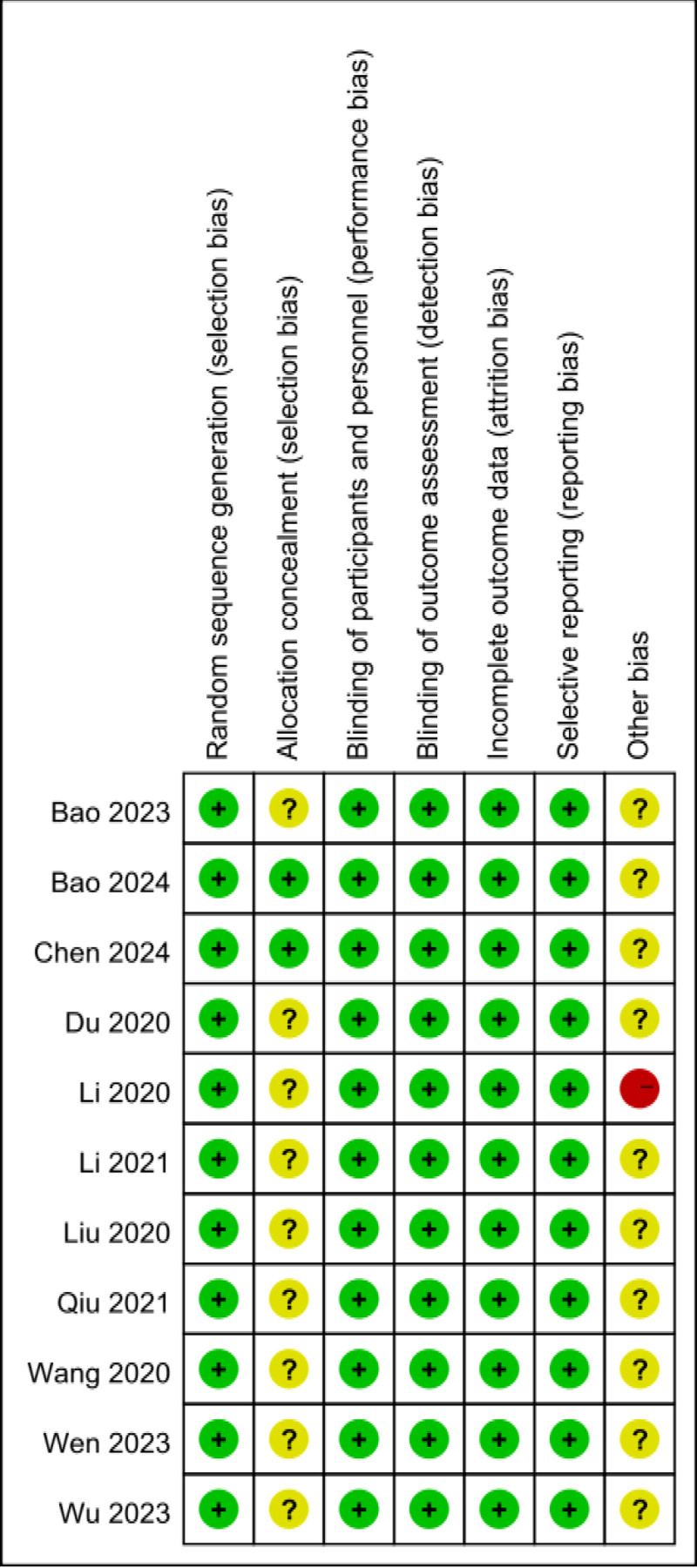
Risk of bias assessment for each study.

The quality of the 11 included studies^[11–21]^ was assessed using the Cochrane Collaboration’s Risk of Bias tool, and the results are presented in Figure 2.

### 3.3. Meta-analysis results

#### 3.3.1. Comparison of effective rate

**Figure 3.**
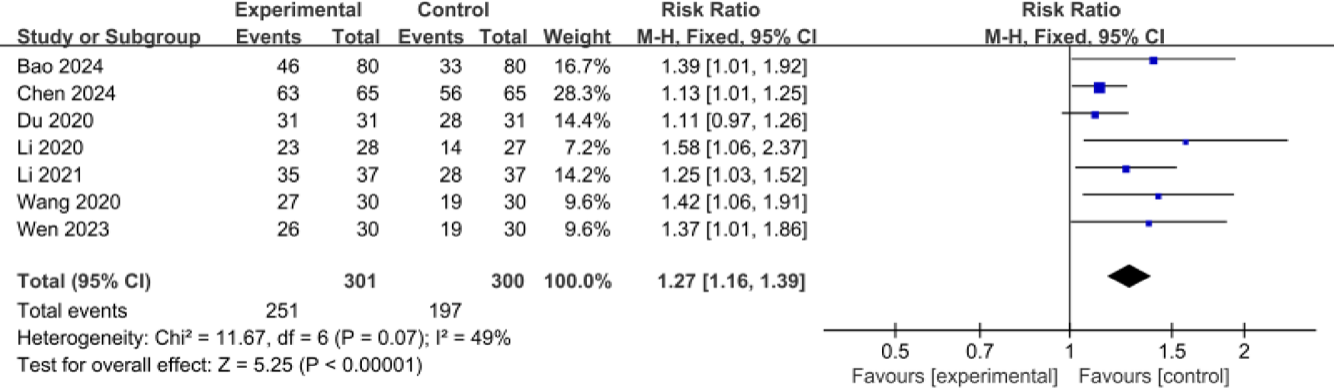
Comparison of effective rate.

The 7 RCTs^[11,14,16,17,19–21]^ included a total of 601 patients (301 patients in the experimental group and 300 patients in the control group); the meta-analysis of the comparison of treatment rate used a fixed-effect model, and there was low heterogeneity between the studies (*I*^2^ = 49%, *P* = .07); the effective rate was 83.39% (251/301) in the experimental group, and 65.67% (197/300) in the control group. The treatment efficiency of the experimental group was higher than that of the control group and the difference was statistically significant (RR = 1.27, 95% confidence interval [CI]: 1.16–1.39, *P* < .001), as shown in Figure 3.

#### 3.3.2. Comparison of adverse event rate

**Figure 4.**
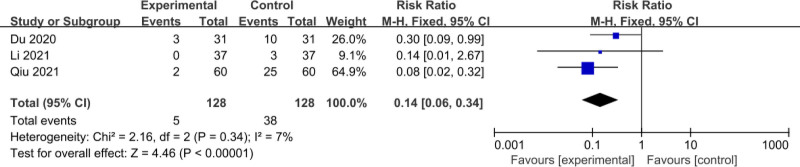
Comparison of adverse event rate.

The 3 RCTs^[11,17,18]^ included a total of 256 patients (128 patients in the experimental group and 128 patients in the control group); the meta-analysis of the comparison of Adverse event rate used a fixed-effect model, and there was no heterogeneity between the studies (*I*^2^ = 7%, *P* = .34); the complication rate was 3.91% (5/128) in the experimental group, and 29.69% (38/128) in the control group. The treatment efficiency of the experimental group was lower than that of the control group and the difference was statistically significant (RR = 0.14, 95% CI: 0.06–0.34, *P* < .001), as shown in Figure 4.

#### 3.3.3. Comparison of pretreatment Hb

**Figure 5.**
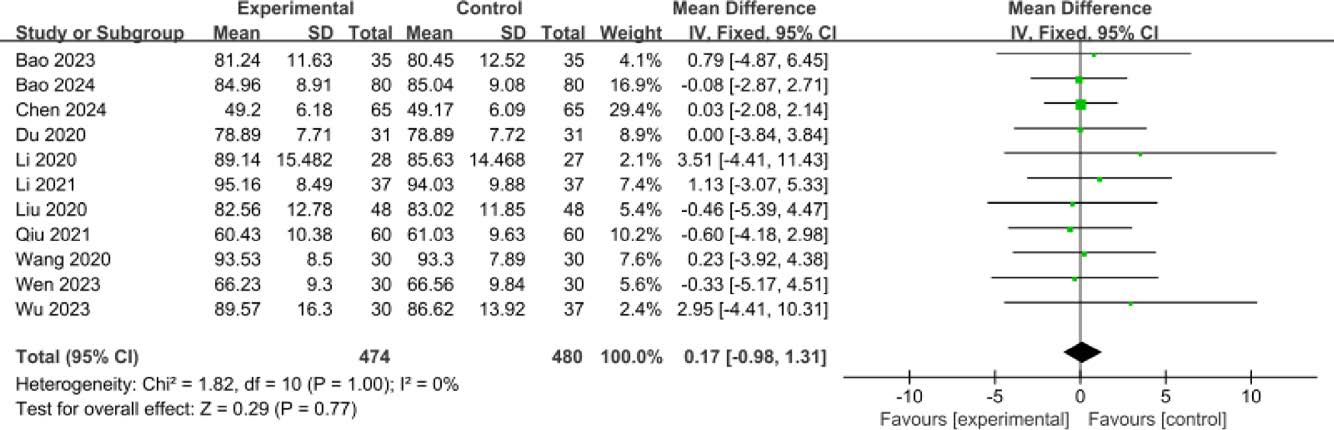
Comparison of pretreatment Hb. Hb = hemoglobin.

The 9 RCTs^[11–14,16,17,19–21]^ included involved a total of 954 patients (474 patients in the experimental group and 480 cases in the control group); meta-analysis of the comparison of pretreatment Hb between the 2 groups, there was no heterogeneity between the studies (*I*^2^ = 0%, *P* = 1.00), and a fixed-effects model was used; there was no statistical significance in the level of pretreatment Hb in the 2 groups (MD = 0.17, 95% CI: ‐0.98 to 1.31, *P* = .77), as shown in Figure 5.

#### 3.3.4. Comparison of posttreatment Hb

**Figure 6.**
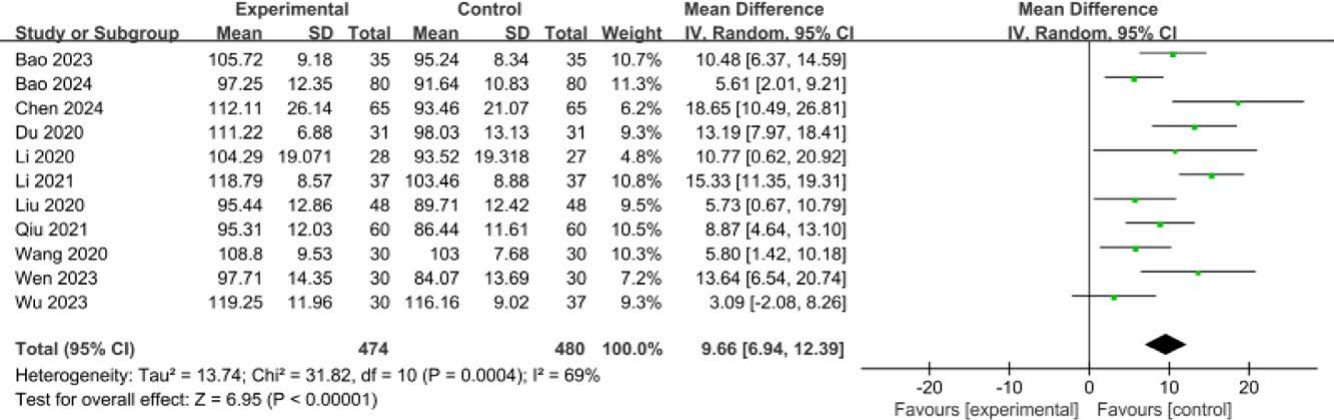
Comparison of posttreatment Hb. Hb = hemoglobin.

The 9 RCTs^[11–14,16,17,19–21]^ included involved a total of 954 patients (474 patients in the experimental group and 480 cases in the control group); meta-analysis of the comparison of posttreatment Hb between the 2 groups, there was middle heterogeneity between the studies (*I*^2^ = 69%, *P* = .0004), and a random-effects model was used; there was a statistically significant posttreatment level of Hb in the 2 groups (MD = 9.66, 95% CI: 6.94–12.39, *P* < .001), as shown in Figure 6.

#### 3.3.5. Comparison of pretreatment RBC

**Figure 7.**
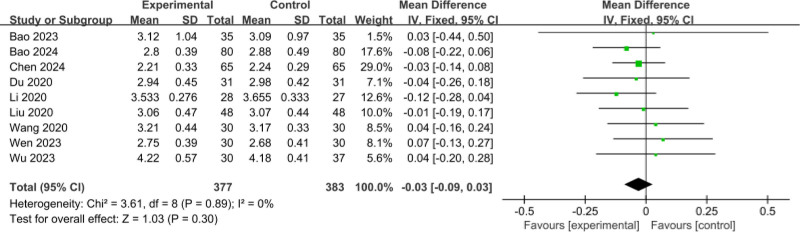
Comparison of pretreatment RBC. RBC = red blood cell.

The 9 included RCTs^[11–16,19–21]^ involved a total of 760 patients (377 patients in the experimental group and 383 patients in the control group); meta-analysis of the comparison of pretreatment RBC, using a fixed-effects model, showed no heterogeneity between studies (*I*^2^ = 0%, *P* = .89); the difference in the comparison of pretreatment RBC was not statistically significant (MD = ‐0.03, 95% CI: ‐0.09 to 0.03, *P* = .30), as shown in Figure 7.

#### 3.3.6. Comparison of posttreatment RBC

**Figure 8.**
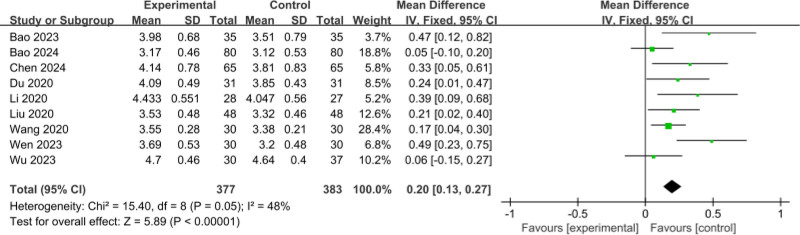
Comparison of posttreatment RBC. RBC = red blood cell.

The 9 included RCTs^[11–16,19–21]^ involved a total of 760 patients (377 patients in the experimental group and 383 patients in the control group); meta-analysis of the comparison of posttreatment RBC was performed with a fixed-effects model, showed low heterogeneity between studies (*I*^2^ = 48%, *P* = .05); and the difference between the comparison of posttreatment RBC was statistically significant (MD = 0.20, 95% CI: 0.13–0.27, *P* < .001), as shown in Figure 8.

#### 3.3.7. Comparison of pretreatment SI

**Figure 9.**
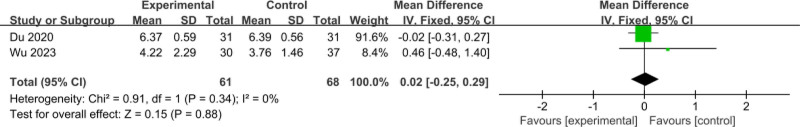
Comparison of pretreatment SI. SI = serum iron.

The 2 included RCTs^[11,12]^ involved a total of 129 patients (61 patients in the experimental group and 68 patients in the control group); meta-analysis of the comparison of pretreatment SI, using a fixed-effects model, showed no heterogeneity between studies (*I*^2^ = 0%, *P* = .34); the difference in the comparison of pretreatment SI was not statistically significant (MD = 0.02, 95% CI: ‐0.25 to 0.29, *P* = .88), as shown in Figure 9.

#### 3.3.8. Comparison of posttreatment SI

**Figure 10.**
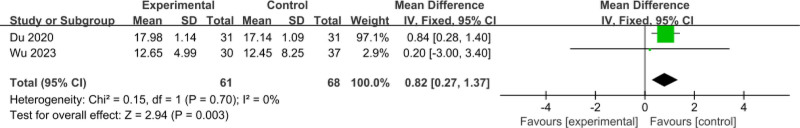
Comparison of posttreatment SI. SI = serum iron.

The 2 included RCTs^[11,12]^ involved a total of 129 patients (61 patients in the experimental group and 68 patients in the control group); meta-analysis of the comparison of posttreatment SI was performed with a fixed-effects model, showed no heterogeneity between studies (*I*^2^ = 0%, *P* = .70); and the difference between the comparison of posttreatment SI was statistically significant (MD = 0.82, 95% CI: 0.27–1.37, *P* = .003), as shown in Figure 10.

#### 3.3.9. Comparison of pretreatment SF

**Figure 11.**
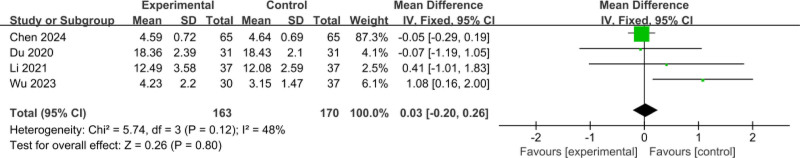
Comparison of pretreatment SF. SF = serum ferritin.

The 4 included RCTs^[11,12,14,17]^ involved a total of 333 patients (163 patients in the experimental group and 170 patients in the control group); meta-analysis of the comparison of pretreatment SF, using a fixed-effects model, showed low heterogeneity between studies (*I*^2^ = 48%, *P* = .12); the difference in the comparison of pretreatment SF was not statistically significant (MD = 0.03, 95% CI: ‐0.20 to 0.26, *P* = .80), as shown in Figure 11.

#### 3.3.10. Comparison of posttreatment SF

**Figure 12.**
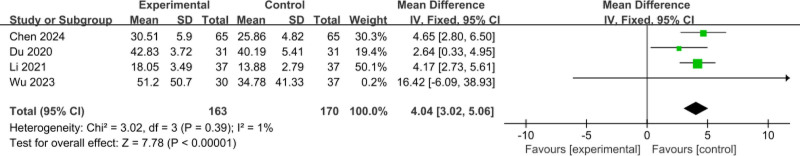
Comparison of posttreatment SF. SF = serum ferritin.

The 4 included RCTs^[11,12,14,17]^ involved a total of 333 patients (163 patients in the experimental group and 170 patients in the control group); meta-analysis of the comparison of posttreatment SF was performed with a fixed-effects model, showed no heterogeneity between studies (*I*^2^ = 1%, *P* = .39); and the difference between the comparison of posttreatment SF was statistically significant (MD = 4.04, 95% CI: 3.02–5.06, *P* < .001), as shown in Figure 12.

#### 3.3.11. Comparison of pretreatment HCT

**Figure 13.**
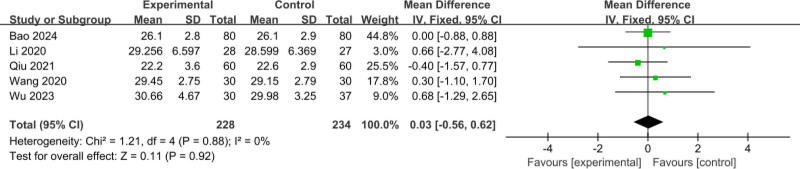
Comparison of pretreatment HCT. HCT = hematocrit.

The 5 included RCTs^[12,18–21]^ involved a total of 462 patients (228 patients in the experimental group and 234 patients in the control group); meta-analysis of the comparison of pretreatment HCT, using a fixed-effects model, showed no heterogeneity between studies (*I*^2^ = 0%, *P* = .88); the difference in the comparison of pretreatment HCT was not statistically significant (MD = 0.03, 95% CI: ‐0.56 to 0.62, *P* = .92), as shown in Figure 13.

#### 3.3.12. Comparison of posttreatment HCT

**Figure 14.**
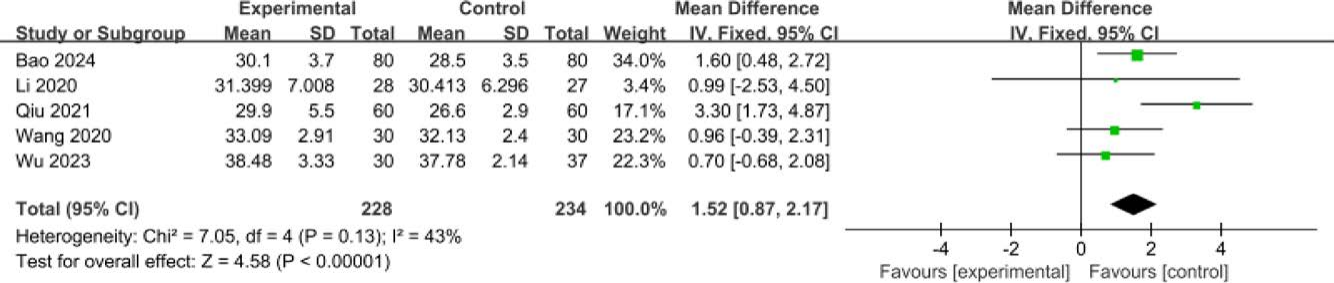
Comparison of posttreatment HCT. HCT = hematocrit.

The 5 included RCTs^[12,18–21]^ involved a total of 462 patients (228 patients in the experimental group and 234 patients in the control group); meta-analysis of the comparison of posttreatment HCT was performed with a fixed-effects model, showed low heterogeneity between studies (*I*^2^ = 43%, *P* = .13); and the difference between the comparison of posttreatment HCT was statistically significant (MD = 1.52, 95% CI: 0.87–2.17, *P* < .001), as shown in Figure 14.

### 3.4. Analysis of publication bias based on effective rate outcomes

**Figure 15.**
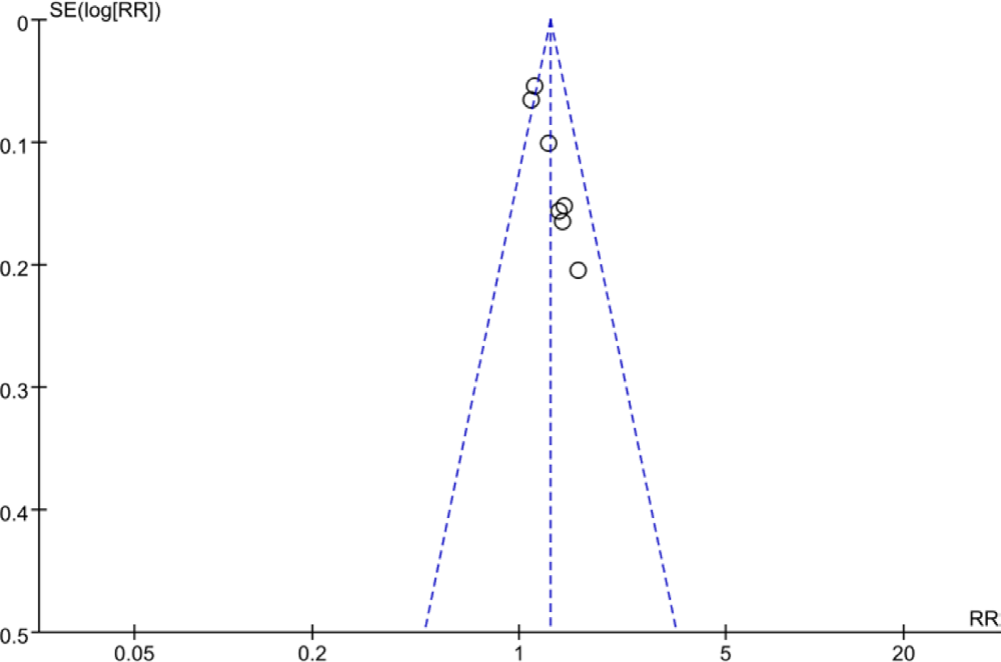
Analysis of publication bias based on effective rate outcomes.

**Figure 16.**
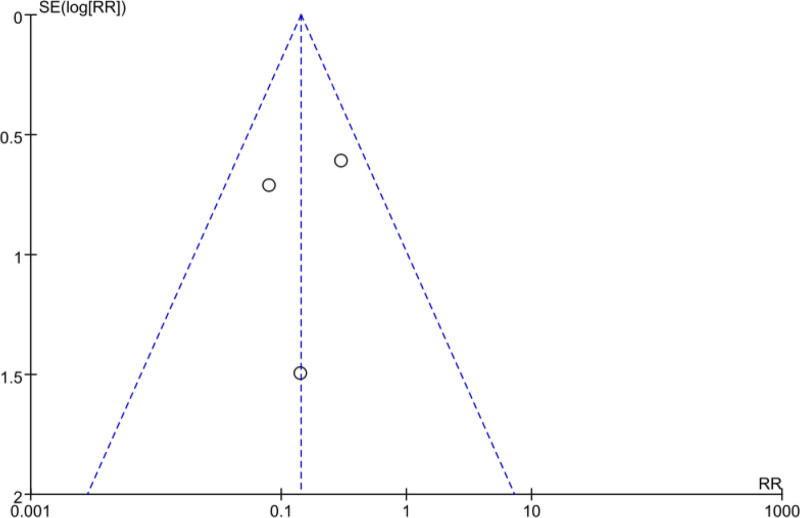
Analysis of publication bias based on adverse event rate outcomes.

By viewing at the scattering points on both sides of the funnel, it can be visualized that they are evenly and symmetrically distributed on both sides of the funnel, which means that there was low probability of publication bias, as shown in Figures 15 and 16.

## 4. Discussion

This meta-analysis incorporated a total of 11 RCTs involving 954 anemia patients. The patients’ type of anemia is aplastic anemia. Gui Pi Decoction can regulate the body’s immune function and reduce the damage of abnormal immunity to hematopoietic stem cells. At the same time, its components can promote the improvement of the hematopoietic microenvironment, stimulate the proliferation and differentiation of hematopoietic stem cells, and then increase the number of peripheral blood cells, playing a therapeutic role in anemia.^[22]^

The meta-analysis results demonstrated that, posttreatment, the improvement in index levels within the experimental group was significantly more remarkable than that in the control group. This implies that treating anemia patients with Guipi Decoction can accelerate the recovery process and exhibits a more favorable overall therapeutic effect. It is worth noting that although Guipi Decoction has a good effect in treating anemia, it is often not used alone. In most cases, it is used in combination with other drugs.

No highly heterogeneous indicators emerged in this meta-analysis. Only 1 indicator, Hb, showed middle heterogeneity, while the rest presented low or no heterogeneity. Therefore, a random-effects model was used only for the hemoglobin indicator, and fixed-effects models were adopted for the others. After carefully examining all the literature, the specific cause of heterogeneity could not be determined. However, it is speculated that this is related to the fact that the drugs used in combination with Guipi Decoction vary across different studies.

There are certain limitations in this meta-analysis: (1) In some of the included studies, Guipi Decoction was used in combination with other drugs. However, it remains unclear how much influence this combination will have on the experimental results; (2) Currently, most of the research on the treatment of anemia with Guipi Decoction is being conducted by Chinese researchers, and there is a lack of foreign RCT literature. Therefore, the conclusions drawn from this meta-study may be controversial.

Using Guipi Decoction to treat anemia patients has demonstrated favorable efficacy and is deserving of application and further promotion. Nevertheless, it is essential to plan randomized controlled studies with larger sample sizes. This will offer more high-quality research foundations for secondary evaluation, thereby enabling a better illustration of the curative effect of Guipi Decoction in treating anemia patients and a more accurate assessment of its promotional value.

## 5. Conclusion

Anemia can cause insufficient oxygen supply to various organs and tissues in the body, leading to a wide range of harms such as dizziness, fatigue, palpitations, weakened immunity, and impacts on growth and development as well as work and daily life. In recent years, traditional Chinese medicine formulas have often been used as a supplement to modern Western medicine drugs in treatment. They often achieve quite good therapeutic effects.^[23]^

Guipi Decoction follows the theory of the mutual generation of qi and blood. Through the combination of qi-tonifying herbs and blood-nourishing herbs, qi can generate blood and blood can carry qi.^[24,25]^ When qi and blood are sufficient, the symptoms of anemia can be improved.

The main ingredients of the prescription are ginseng, atractylodes macrocephala and astragalus membranaceus. Ginseng contains components such as ginsenosides. It can stimulate hematopoietic organs, promote the proliferation and differentiation of bone marrow hematopoietic stem cells, and increase the production of blood cells such as red blood cells and white blood cells. Moreover, it can enhance the body’s immunity and the resistance to diseases, creating favorable conditions for the body’s recovery.^[26–28]^ Astragalus membranaceus mainly contains components like astragalus polysaccharides and flavonoids. It can promote the secretion of hematopoietic growth factors in the hematopoietic microenvironment, stimulate the proliferation of hematopoietic stem cells, increase the hemoglobin content, regulate the immune system, and improve the overall state of the body, which helps to alleviate the symptoms of anemia.^[29]^ Atractylodes macrocephala contains volatile oils, atractylodes polysaccharides, etc. It can regulate the gastrointestinal function, enhance the transportation and transformation ability of the spleen and stomach, enabling the body to better absorb nutrients from food, providing sufficient raw materials for the generation of qi and blood.^[30]^ Additionally, it can boost the immunity and assist in improving anemia.

The rational use of Guipi Decoction can rapidly alleviate the symptoms of anemia patients, demonstrating definite curative effects and improving their quality of life. It is essential to have a proper understanding of the efficacy of Guipi Decoction, actively conduct relevant experiments, and continuously optimize its application methods. This is expected to contribute to the health of anemia patients worldwide.

## Author contributions

**Writing – original draft:** Xueyan Lv.

**Writing – review & editing:** Dongmei Wang, Xiangni Zou.
